# Notes of Three Cases of the Administration of Chloroform to Children

**Published:** 1851-07

**Authors:** Geo. N. Burwell


ART. VI.—*Notes of Three Cases of the administration of Chloroform to Children.* Reported by GEO. N. BURWELL, M. D. Read at the meeting of the Buffalo City Medical Association, June 17, 1851.

CASE 1. *Strangulated Hernia.*—I was called on June 4th, 1851, to see a child between seventeen and eighteen months old, with strangulated hernia of the right side. The hernia was congenital, and had been frequently down in the first months of the child's life. It had not been down before for three months; had never before been strangulated. It had got down about 2 o'clock, and Dr. Nott was called to reduce it. He succeeded very quickly, but while putting on the truss, the boy crying considerably, it suddenly slipped down again, and resisted all farther efforts to reduce it. The Doctor tried faithfully for two hours or more, and then desisted until I could arrive. While thus waiting, cloths wrung out in cold water were kept diligently applied. I saw the child at seven o'clock. The child was lying quietly in its mother's lap, apparently asleep, and had thus lain for two hours, exhausted, probably, by previous crying. There had been occasional vomiting—extremities cool; face pale. The hernia was about the size of three fingers of a man's hand when laid in closely together. The child began to cry and worry as soon as it was touched. Twelve or fifteen drops of chloroform were poured on to a handkerchief and held to the child's nostrils. This quantity was renewed as often as it had evaporated. The child made considerable efforts to resist the breathing of it, but its use was persevered in until complete anaesthesia was produced. This was indicated by a discontinuance of all resistance to its use; free, deep regular inspirations, in the place of irregular ones noticed before, increased pallor of face, and general muscular relaxation. The pulse was not sensibly altered in frequency, remaining at about 100, but it had, apparently, more fullness and force. There was no stertorous breathing at any time.

The use of the chloroform was of course immediately discontinued, and taxis again attempted, and after four or five minutes I had the pleasure of feeling the protruded bowel slip suddenly into the abdomen. Once during this time it was necessary to administer more chloroform for a few inspirations, he showing some restlessness. He lay quietly asleep while the truss was being applied, and was then laid on the bed, and continued to sleep while I remained, some ten or fifteen minutes. He had no further trouble from either the hernia or the chloroform.

CASE 2. *Fractured Femur.*—I gave chloroform, in April last, to a boy four years old, for the purpose of reducing a fractured femur. The child was much frightened, and would cry inordinately, even on turning down the cloths to look at the limb; and there was no controlling his screams when we attempted to handle it. I preferred to administer the chloroform to enduring his cries. He took it reluctantly; but by persevering with it, as in the case just related, he soon became insensible, and we were enabled to examine the limb, make sure the diagnosis by producing crepitus, and apply the splints and bandages at our leisure, without a cry or particle of resistance on his part. When any restlessness was exhibited, more chloroform was instantly administered. After being placed in bed, he soon came to himself. His breathing during its administration was slower and heavier than when awake, but without any stertor. His pulse remained natural. He was about half an hour under its influence. I gave it to him on the first re-application of the dressings; afterwards I was able to get along well enough without it.

Case 3. *Extraction of a Kernel of Coffee from the Ear.*— This boy was between four and five years old. He had one afternoon pushed a grain of coffee far into his right ear. It remained there until the next forenoon, when he was brought to my office to have it taken out. It had become much swollen, and filled entirely the meatus. All efforts to hold the child so it could be reached with a delicate pair of forceps, proved unavailing, although three persons attempted it a portion of the time. With their best efforts he would writhe himself about, raise and fall his shoulder, twist his head from side to side, so the forceps could not be safely introduced.

Immediately on the chloroform producing its effect, his father laid him across his knees, a perfectly passive being. The kernel was then easily seized hold of and extracted. The boy, exhausted by his previous efforts, lay quietly asleep for fifteen minutes, and then suddenly awoke, and sprang from his father's lap, with the exclamation of "Father, I want to go home," without seeming to care in the least about the kernel being out of his ear.

I wish, also, to mention to the Association, that I have twice administered chloroform to ease the pains of the dying. In the first case, death resulted from the pressure of an enormous ovarian tumour and dropsy. The patient's sufferings were intense during the last two days of her life, except as relieved by morphine and chloroform. The chloroform was left with her to take as she chose, due caution being given to avoid an overdose.

The second case was one of acute Peritonitis. After passing some days without any acute suffering, the patient was, on the morning of her death, taken with very acute pain so she constantly screamed with agony. It was entirely evident from her pulse and general appearance, that she was already dying; and as the only way to afford prompt relief, I administered chloroform.

The relief was great while she continued under its influence. Occasionally she would get enough to cause a temporary loss of consciousness, but never more than momentarily so. She continued its use until she died, some four hours.

